# Adherence to Glaucoma Medications Over 12 Months in Two US Community Pharmacy Chains

**DOI:** 10.3390/jcm5090079

**Published:** 2016-09-07

**Authors:** Michael Feehan, Mark A. Munger, Daniel K. Cooper, Kyle T. Hess, Richard Durante, Gregory J. Jones, Jaime Montuoro, Margaux A. Morrison, Daniel Clegg, Alan S. Crandall, Margaret M. DeAngelis

**Affiliations:** 1 Department of Pharmacotherapy, College of Pharmacy, University of Utah, Salt Lake City, UT 84112, USA; mark.munger@hsc.utah.edu; 2 Hall & Partners, New York, NY 10022, USA; danny.cooper@themodellers.com (D.K.C.); kylethess@gmail.com (K.T.H.); 3 Marketing and Planning Systems (MaPS), a Division of Millward Brown Analytics, Boston, MA 02116, USA; rdurante@mapsnet.com; 4 Harmon City, Inc., West Valley City, UT 84120, USA; gregjones@harmonygrocery.com; 5 Smith’s Food & Drug Centers, Inc., Salt Lake City, UT 84104, USA; jaime.montuoro@sfdc.com; 6 Department of Ophthalmology & Visual Sciences, Moran Eye Center, University of Utah School of Medicine, Salt Lake City, UT 84132, USA; margaux.morrison@utah.edu (M.A.M.); doc1@utah.edu (D.C.); alan.crandall@hsc.utah.edu (A.S.C.)

**Keywords:** adherence, glaucoma, pharmacy

## Abstract

This study determined the degree of adherence to medications for glaucoma among patients refilling prescriptions in community pharmacies. Methods: Data abstracted from the dispensing records for 3615 adult patients (18 years or older, predominantly over 45) receiving glaucoma medications from two retail pharmacy chains (64 stores in total) were analyzed. From a 24-month historic data capture period, the 12-month levels of adherence were determined using standard metrics, the proportion of days covered (PDC) and the medication possession ratio (MPR). The overall 12-month mean PDC was only 57%, and the mean MPR was 71%. Using a criterion by which 80% coverage was considered satisfactory adherence, only 30% had satisfactory overall 12-month PDC coverage, and only 37% had satisfactory overall 12-month MPR coverage. Refill adherence increased with age and was highest in the 65-and-older age group (*p* < 0.001). Differential adherence was found across medication classes, with the highest satisfactory coverage seen for those taking alpha2-adrenergic agonists (PDC = 36.0%; MPR = 47.6%) down to those taking direct cholinergic agonists (PDC = 25.0%; MPR = 31.2%) and combination products (PDC = 22.7%; MPR = 31.0%). Adherence to glaucoma medications in the community setting, as measured by pharmacy refill data, is very poor and represents a critical target for intervention. Community pharmacists are well positioned to monitor and reinforce adherence in this population.

## 1. Introduction

Medication non-adherence is recognized as one of the most important and costly worldwide healthcare problems in the 21st century [1]. In the United States, an estimated $100–290 billion in preventable costs can be realized by improving the 30%–50% adult non-adherence rate to chronic medications [2]. Open-angle glaucoma (the most common form affecting 90% of glaucoma cases) is the leading cause of irreversible blindness in the US and worldwide, following cataract, which is reversible [3]. The only proven method of slowing glaucoma is to lower intraocular pressure (IOP) with daily medications for an indefinite period [4]. Ocular disorders such as glaucoma are recognized to be amongst the top five conditions, where progression is related to poor patient adherence [5]. Patient non-adherence to glaucoma medication is highly probable, with rates similar to the non-adherence rates with oral medications for other chronic asymptomatic conditions such as hypertension and hypercholesterolemia [6]. Non-adherence to glaucoma medications has been estimated to range from 24% to 59%; furthermore, in a study of claims data, 50% of patients stopped taking their medications within six months, and only 37% had their prescriptions filled three years after initial dispensing [4]. According to Schwartz and Quigley, research brings the “unwelcome conclusion that persistence with initial glaucoma medication is as low as 33%–39% at one year” [7].

Since reduction in IOP with ocular hypotensive agents is effective in slowing the progression of glaucoma, non-adherence could be considered a risk factor for irreversible vision loss [5,8,9]. Importantly, non-adherence to treatment has been shown to contribute significantly to disease progression and avoidable vision loss [9,10,11,12,13]. Non-adherent patients have been shown to have weaker beliefs in the effectiveness of their glaucoma medications, more concerns about those medications, and poorer quality of life (QoL) [14].

Measuring adherence to medications in general can be a challenge. Direct measures of adherence (e.g., drug concentrations or metabolites in blood) are objective but can be expensive to use routinely and over extended periods of time outside the research setting. In contrast, self-report data is simple and inexpensive to gather but can be biased through recall bias and socially desirable responding, and many studies have shown that patients with glaucoma consistently over-estimate their level of adherence [15]. Electronic monitoring (e.g., of the opening of a unit) is more expensive and is compromised by the fact that the patient may not have used the dose as directed once opened. There is also evidence that glaucoma medication adherence improves immediately preceding and following scheduled doctors’ visits [4,7,15]. Using recordings of when an eye medication dispenser was removed from a transparent bottle with an electronic counter top, around 80% of patients were considered adherent [16]; however, glaucoma adherence agreement between technological dosing aid records, patient reports, and physician assessments can be very poor [6,7].

Pharmacy claims data provide objective medication re-fill adherence metrics, though challenges can exist in this approach through varying definitions and algorithms used to define poor adherence [17]. Nevertheless, these measures are robust enough to be used to validate well-recognized self-report measures [18,19]. In a study of 13,956 claims from a managed care pharmacy database, the mean medication possession ratio (MPR) was only 0.64 [16]. The use of pharmacy claims data is also useful in that it can provide immediate feedback and opportunity for intervention by the dispensing pharmacists, frequently involved as intervention providers in other disease state intervention studies [20,21,22,23,24].

The aim of the present study was to determine the degree of adherence to medications for glaucoma in two community pharmacy chains, as measured by refill rates across medication classes in two representative longitudinal pharmacy dispensing databases.

## 2. Experimental Section

### 2.1. Sample and Records

Glaucoma medication refill data were abstracted from the dispensing databases of two community pharmacy chains comprising 64 retail pharmacies in Utah, USA. De-identified data were abstracted such that neither the patient nor the prescriber could be identified by the research team. A total of 24 months of historical records were evaluated, encompassing the period 1 May 2013 through 31 April 2015. These data comprised 69,421 prescription records for 32,564 patients. The age and sex of patients is recorded in these data, but not ethnicity. Analyses were conducted on the records of 3615 adult patients (aged 18 years or older), for whom a glaucoma eye-drop medication was dispensed in the 24-month period and who had a minimum of 12-month potential coverage (365 days) from the date of their first fill of a medication in that exposure window.

### 2.2. Glaucoma Medications

All patients had been dispensed a glaucoma medication as listed in the Facts and Comparisons database [25]. These medications included prostaglandin analogs, beta receptor antagonists, carbonic anhydrase inhibitors, direct and indirect cholinergic agonists, alpha2-adrenergic agonists, and combination products (two medications in the same eye-drop).

### 2.3. Adherence Estimation

Adherence metrics were calculated for each patient over 365 days. Two calculations were made based on the days of medication prescribed in each dispensing refill, the proportion of days covered (PDC) [26] and the medication possession ratio (MPR) [27]. The calculations of these metrics and the degree of concordance between them have been described in detail by the Pharmacy Quality Alliance [28]. In our analyses, the PDC represented the number of days for which a patient had a medication over the period from the first fill within the year of exposure, and the MPR represented the days of medication availability over the number of days from the first dispensing to the end of the coverage of the most recent refill. As has been reported extensively in the literature, adherence was considered unsatisfactory if medications were not available at least 80% of the time. This approach with a community pharmacy database has been used previously by the authors in a study of adherence medications for asthma [29].

## 3. Results

The records analyzed came from 3615 unique adult patients. The majority were predominately women (57%), and, as glaucoma is a disease of older age, the majority were over the age of 65 (69%), compared with 23% in those aged 45–64 years and only 8% in those aged 18–44 years (see Table 1). All patients were taking one prescription medication within each of the classes: prostaglandin analogues (*n* = 1898; 52.5%), combination products (*n* = 828; 22.9%), beta receptor antagonists (*n* = 438; 12.1%), alpha2-adrenergic agonists (*n* = 168; 4.6%), and direct cholinergic agonists (*n* = 16; 0.4%).

The adherence rates over 365 days are shown in Table 1. The average PDC was 57%, and the average MDC was 71%. Overall, 70% of patients had unsatisfactory PDC (<80%), and 63% had unsatisfactory MPR (<80%).

No significant sex differences were found in the average PDC and MPR levels (*p* > 0.05), nor across the percentages of each gender, with satisfactory levels of PDC and MPR coverage (*p* > 0.05). Statistically significant improvements in adherence were seen with older age in the average PDC and MPR levels (*p* < 0.001), and the percentage with satisfactory adherence on each metric. The PDC proportions with satisfactory adherence rose from 9% (ages 18–44) to 19% (ages 45–64) and to 35% for those aged 65 or older (*p* < 0.001). Similarly, on MPR, the percentage with satisfactory adherence rose from 13% (ages 18–44) to 27% (ages 45–64) and to a high of 43% for those aged 65 years or older (*p* < 0.001).

There was meaningful variation in adherence by class. Those taking alpha2-adrenergic agonists had the highest levels of satisfactory coverage (PDC = 36.0%; MPR = 47.6%) followed by those taking carbonic anhydrase inhibitors (PDC = 34.5%; MPR = 42.9%), beta receptor antagonists (PDC = 32.9%; MPR = 40.2%), and prostaglandin analogues (PDC = 30.6%; MPR = 37.1%). The lowest levels of satisfactory coverage were evident for those taking direct cholinergic agonists (PDC = 25.0%; MPR = 31.2%) and combination products (PDC = 22.7%; MPR = 31.0%).

## 4. Discussion

This analysis of refill data from community pharmacies across the state of Utah indicated very high 12-month levels of unsatisfactory adherence (given the risk of blindness from glaucoma) for both women and men, though the levels improved with age. On the MPR, the mean percentage was 71%, which is similar to the mean percentage of 64% reported by Freidman et al. [16]. It is clear that patients with glaucoma have inadequate adherence using this measure, with patients only having medication available around two-thirds of the time. The proportion of patients with overall ‘satisfactory’ 80% coverage in this study is disheartening, ranging from 30% for the PDC to 37% for the MPR. These data confirm the potential for greater morbidity in terms of lost visual acuity, poorer quality of life, and greater cost burden of this disease unless adherence to glaucoma medications is improved. It is interesting to note that adherence in terms of refills was higher in the over-65 age group despite an increased likelihood of medication burden through multiple co-morbidities. This older age group may be refilling scripts more consistently given the potential for better insurance coverage, and the driving influence of the severity of other diseases for which they receive medications which may increase adherence motivation [30]. It still remains a question if they are achieving the benefits of satisfactory dosages given the oft-cited challenges of effectively administering eye-drops [15].

While there was variation in the degree to which each medication class had satisfactory levels of coverage, only 50% of patients had satisfactory coverage with the most-adherent agents (the alpha-2 antagonists). Further research is warranted to understand why certain patients receive different IOP lowering agents than others, and critically, whether the greater adherence rates for some classes result in different clinical visual functioning outcomes over time; given non-adherence to treatment has been shown to contribute significantly to disease progression and avoidable vision loss [9,10,11,12].

A limitation of this study is that it is an analysis of refill data, and it is possible that patients may have refilled a script elsewhere. Patients may also have received periods of samples from prescribing physicians, which may lead to under-reporting in dispensing data [19]. These may inflate the level of low adherence; however, given the stable nature of the Utah population, and the level of consistent patronage seen in the partnering pharmacies based in routinely used grocery stores, this bias is likely to be small. Given the challenges of eye drops use, especially in the elderly, even those patients that have satisfactory coverage of medications may have sub-optimal delivery and adequate dosing. It is not possible to determine what psychological and socioeconomic factors may be contributing to low levels of satisfactory adherence in this population, without recourse to primary patient-centric research. These data highlight the need for studies into why these levels of adherence are so low and into the development of empirically justified interventions to improve glaucoma medication adherence in the community setting.

It is the goal of this research team to conduct further research with patients from these pharmacy settings to determine why patients are less adherent in a disease state that could leave them blind. Numerous factors are commonly associated with non-adherence to medications in general, including complex drug regimens or high pill burdens, low health literacy, unclear or misunderstood instructions, impaired cognition, lack of social support, forgetfulness, concerns about side effects, cost, low perceived need or efficacy, and limited patient engagement [30]. Robin and Grover assert that adherence to eye drops poses a set of unique challenges versus oral medications used for other conditions [15]. Eye drops require manual dexterity, eye-hand coordination, and good vision. Studies have shown that adherence to eye-drops also falls off when other medications are added to the regimen. Common reasons cited for non-adherence to glaucoma medications include forgetfulness, inability to use eye drops accurately, inconvenience, or frequent dosing, medication cost and side effects, travel and scheduling, denial, perceived risk of blindness, and depression [4,7,15]. A taxonomy is listed of factors of 71 situational or environmental factors (49%), regimen factors (32%), patient factors (16%), and provider factors (3%) that may be associated with non-adherence to glaucoma medications [13]. Given no primary interview data were available in our study, it is not possible to ascertain in this population why patients were non-adherent, or, for example, why the older age group patients had higher refill rates.

In general, the successes of medication adherence interventions across disease states have been relatively low, though even small improvements could result in substantial cost savings [31]. It appears that multifaceted interventions may hold the most promise, including those that involve medication simplification, addressing barriers by improvement in convenience, provision of information, counselling, refill reminders, and self-monitoring and follow-up, especially when tailored to the individual [31]. In glaucoma, such general approaches have been shown to improve adherence, including educating the patient, simplifying treatment regimens, involving care-providers, and customization to the patient’s lifestyle. More targeted approaches include education about adherence per se [15]. Using a daily text-message reminder-based intervention significantly improved glaucoma adherence rates from 54% to 64% in an intent-to-treat analysis [16]. Studies that have targeted patient beliefs have been effective at improving adherence; interventions directed only at providing glaucoma education, however, have failed to demonstrate significant improvements in adherence [9]. As pharmacists have access to dispensing data from which they can monitor refills and have more regular contact with patients than prescribing physicians, they are well positioned to offer medication management counselling to patients to enhance adherence. The engagement of pharmacists in a team-based approach with prescribing physicians, to monitor refill data and support individualized education plans for patients, is in line with recent empirically based recommendations to improve glaucoma adherence [32].

## 5. Conclusions

This study has demonstrated the unacceptably high levels of glaucoma medication non-adherence as measured through refill data in settings where patients receive their daily maintenance medications, and pharmacists are available to serve as educators and facilitators for adherence. In order to design better multifaceted interventions to remedy this situation, it is necessary to ground change techniques in empirical data. We therefore aim to interview patients from these community pharmacies and reconcile their self-reports of adherence with objective refill database data and model the psychosocial, attitudinal, socioeconomic, and genetic predisposing factors that may contribute to this problem in order to identify targets for change that can be modified in pharmacy-centric interventions wherever these patients are filling their prescriptions.

## Figures and Tables

**Table 1:** Degree of adherence indicated by refill data, the proportion of days covered (PDC) and medicine possession ratio (MPR), by sex and age.

		Sex				Age (Years)				
	Total	Female	Male	Statistic	*P*-Value	18–44	45–64	65 and Over	Statistic	*P*-Value
	(*n* = 3615)	(*n* = 2074)	(*n* = 1541)			(*n* = 275)	(*n* = 843)	(*n* = 2497)		
**Proportion of Days Covered (PDC)**										
Mean percent PDC (SD *)	57 (35)	57 (35)	56 (35)	*t* = 0.85	0.394	29 (29)	48 (33)	62 (34)	*F* = 164.8	<0.001
Number with satisfactory PDC ^1^ (%)	1070 (30)	622 (30)	448 (29)	χ^2^ = 0.36	0.550	26 (9)	164(19)	880 (35)	χ^2^ = 133.3	<0.001
**Medicine Possession Ratio (MPR)**										
Mean percent MPR (SD *)	71 (59)	72 (59)	71 (60)	*t* = 0.19	0.849	34 (42)	58 (53)	80 (61)	*F* = 108.1	<0.001
Number with satisfactory MPR^1^ (%)	1342 (37)	783 (38)	559 (36)	χ^2^ = 0.83	0.363	36 (13)	229 (27)	1077 (43)	χ^2^ = 142.5	<0.001

* SD, standard deviation; ^1^ Satisfactory PDC and MPR defined as ≥ 80%.
